# Research on the Performance of Human Capital at Different Organizational Levels of Pharmaceutical Corporations: Moderation of Informal Relational Capital

**DOI:** 10.3390/ijerph18157852

**Published:** 2021-07-24

**Authors:** Shenglei Pi, Kuei-Feng Chang, I-Tung Shih

**Affiliations:** 1 Guangzhou Higher Education Mega Center, Department of Strategies and Innovation, School of Management, Guangzhou University, 230 Wai Huan Xi Road, Guangzhou 510006, China; shenglei@gzhu.edu.cn; 2 Guangzhou Higher Education Mega Center, Department of Marketing Management, School of Management, Guangzhou University, 230 Wai Huan Xi Road, Guangzhou 510006, China; 3 Department of Business Administration, Chaoyang University of Technology, 168 Jifeng E. Rd., Wufeng District, Taichung 413310, Taiwan; shih1238@gm.cyut.edu.tw

**Keywords:** human capital in different levels, intellectual capital, informal relational capital, resources allocation

## Abstract

As intellectual capital is considered an important strategic resource in knowledge-intensive industries, such as the health and pharmaceutical industries, scholars have developed a deeper understanding of the human capital at different levels of organizations and its interaction with relational capital from informal institutional stakeholders. This study focused on the role of human capital at different levels of pharmaceutical corporations and the orchestration of human capital at different levels with informal relational capital. By using data regarding Chinese pharmaceutical listed companies from 2001 to 2017, this study found that (1) human capital at the employee level exerted an inverted U-shaped effect on a pharmaceutical firm’s performance, which was negatively moderated by informal institutional relational capital, and (2) based on the upper echelons theory, human capital at the executive level had a monotonic positive impact on a pharmaceutical firm’s performance but was negatively moderated by informal relational capital. We found two arrangements that facilitate the orchestration of intellectual capital components to gain optimal distinctiveness and performance.

## 1. Introduction

Knowledge-intensive sectors, such as the pharmaceutical and health industries, create value mainly based upon the integration of intellectual capital. Intellectual capital covers all the knowledge that an enterprise can access as an organization [1]. Some scholars [2,3,4,5] categorize intellectual capital into three components, namely, human capital, structural (organizational) capital, and relational (social) capital. Researchers have conducted various studies on how these components influence performance [6,7,8,9,10,11,12] among all external and internal resources and legitimacies for performance. Many researchers presume that these three components are independent of each other such that their impacts on performance can be simply summed up as their total impact on the innovative strategies of an enterprise [10,13,14].

Previous studies focusing on human and relational capital have presented different and even contradictory effects on performance. Some scholars prefer to represent human capital as the executives or CEOs [15], while some consider human capital as all employees of an enterprise, regardless of their positions [16,17]. Neither of these two viewpoints takes into account the heterogeneity of human resources at different levels of an organization. Previous studies on human capital failed to reveal the various effects of human capital at different organizational levels on an organization’s performance and even failed to discuss any possible coordination or orchestration between human capital at the executive and employee levels for the sake of optimal performance. Additionally, in recent years, scholars have expanded the original definition of relational capital [18] from customer relational capital to relational capital that covers all stakeholders [19]. Additionally, a new agenda of informal relational capital has been introduced into organizational research [20,21]. Different stakeholders have different requirements of legitimacies for focal firms [22]. Previous studies failed to explore the diverse influences of different types of relational capital on performance, the interplay between relational capital and human capital of a specific level, and their mechanism if they exists.

From the perspective of optimal distinctiveness theory, the internal heterogeneity of an enterprise needs to be integrated with external legitimacies to achieve optimal distinctiveness and performance [23]. The human capital at different levels may have various impacts on performance that are contingent on different informal relational capital. In this way, optimal distinctiveness can be achieved by combining internal human capital and external relational capital. Furthermore, human capital at different organizational levels has different impacts on performance. Thus, there are dynamics in the combination of specific forms of human capital and relational capital.

By using data regarding Chinese pharmaceutical listed companies from 2001 to 2017, this study explored the various impacts of human capital at different levels of firms on the performance and the moderating effects of relational capital under an informal institutional context. This research revealed two groups of orchestration between the two major components of intellectual capital in pharmaceutical firms: (1) human capital at both levels exerted an impact on performance in the same direction at first and then in different directions, implicating the need to allocate human capital at both levels to achieve optimal performance, and (2) with a change in informal relational capital, an enterprise needs to dynamically orchestrate both human capital levels for the sake of optimal performance.

## 2. Theoretical Perspectives

### 2.1. Optimal Distinctiveness and Intellectual Capital

Optimal distinctiveness was originally developed by [24] in social psychology and applied in various fields to investigate the equilibrium between being different and conforming to legitimacies [25]. Diversity without legitimacies merely appears “odd” to stakeholders instead of representing competitiveness [24]. A firm should achieve a distinctive identity of normalized legitimacy or be the “same and different at the same time.” Since then, studies have emerged and addressed the factors and mechanisms that are involved with reaching optimal distinctiveness in an organization [26]. Researchers suggested that managers establish appropriate heterogeneous advantages, which are developed and supported by institutional legitimacies, to promote the distinctive advantages of a firm [25,27,28].

According to optimal distinctiveness theory, internal heterogeneous resources only lead to competitive advantages and firm performance by orchestrating external social–relational resources properly [28,29,30]. In other words, optimal distinctiveness can only be achieved by properly orchestrating internal and external resources. Thus, there are two major ways of achieving distinctiveness between the heterogeneous resources in a firm and external stakeholders’ legitimacies or relational resources. First, there is a “scale boundary” in heterogeneous resources when external legitimacies are insufficient, but the combination of internal resources and external relational resources will help to break through this boundary and improve performance [28]. Second, external legitimacies restrain the effect of internal resources on performance and together they form one of the highest performance values [27].

Prior research on intellectual capital was mostly carried out based on the summation of the three components of intellectual capital or the interplay between the three components and performance. The interactions between human capital and relational capital [7,13], between structural capital and relational capital [31], and between human capital and structural capital [6,16] were the topics of recent studies, but few of these studies examined the interaction between the three components of intellectual capital and performance as a whole and tried to explore the influential mechanism between them. The three components of intellectual capital, namely, human capital, relational capital, and structural capital, represent internal heterogeneous resources, external institutional resources, and organizational resources, respectively [19,32]. Moreover, research on the relationship between structural capital and the two other kinds of capital is very limited. The impacts of the three components of intellectual capital on performance are not independent of each other or interactive but are based on one or several types of orchestration. However, only a few studies have tried to explore the orchestration of three components from the perspective of optimal distinctiveness.

### 2.2. Human Capital at Executive and Employee Levels from the Perspective of Optimal Distinctiveness

Human capital is composed of the ability, knowledge, working attitude, and skills of employees that are involved in an enterprise [33]. The role of human capital in intellectual capital has been discussed, and some scholars have even directly substituted intellectual capital with human capital [34,35,36]. Current research on human capital focuses on two different levels, namely, executives and employees. The studies that approach executive human capital emphasize the importance of executives’ decisions, managerial knowledge, and skills on enterprises from the perspective of upper echelons theory [32,37,38]. Yan et al. [32] showed that the knowledge, skills, and strategic cognition of senior executives were important factors that dominated and controlled the strategic behavior of enterprises. Rossi et al. [13] found that managers and senior human resources validate practical actions that distinguish firms. Scholars who focused on human capital involving employees underlined the impact of total factor productivity, education, training [14,17], and knowledge acquisition [39] on the competitive advantages of enterprises and industries.

Upper echelons theory shows that executives help enterprises build heterogeneous advantages and improve performance, mainly through strategic decision making in an uncertain environment [37,40]. Therefore, the senior management team integrates diverse expertise and experience to help enterprises build heterogeneous advantages despite a possible conflict in the cognition of the senior management team and decision-making difficulties [41]. Therefore, human capital is an important decision-making tool for ensuring that enterprises build and give full play to their advantages in a sustainable manner from the perspective of upper echelons theory. Thus, Hypothesis 1a was proposed:

**Hypothesis** **1a** **(H1a).** *A firm’s executive human capital has a positive influence on its performance.*

Economics and management scholars have been studying the labor force and productivity of employees [42]. Their education level, learning ability, and absorptive capacity determine the overall learning and absorptive capability of an organization [17,43]. However, the improvement in employees’ learning and absorptive ability will lead an organization to boost its knowledge generation, and whether the knowledge can be transformed into market competitiveness depends on the organization capacity [19,44]. When the organizational learning ability is insufficient, a mere increase in human capital at the employee level may effectively improve the knowledge management and absorptive abilities of enterprises and, thus, improve their performance. With the increase in internal knowledge reservation, the burden of employees’ learning and digesting of redundant internal knowledge is also increased, which may reduce the marginal return of organizational learning [45]. Meanwhile, the organization itself also has a scale boundary. Once the scale of employees exceeds the boundary of the scale economy and enters the diseconomies of scale, an increase in human capital is not conducive to performance growth, no matter how much human capital there is. Consequently, without taking into consideration other factors, human capital will first promote performance growth and then gradually weaken the organizational performance, along with the increase in executive human capital. Thus, Hypothesis 1b was proposed:

**Hypothesis** **1b** **(H1b).** *Employee capital can change the U-shaped influence on performance.*

### 2.3. Social Capital from Formal and Informal Stakeholders

The institutional literature considers institutions such as laws, rules, and regulations as formal institutions [46], while informal institutions include cultures, norms, and values [44,47]. In the formal context, firms engage in businesses that are compliant with the laws and regulations [48], while in the informal context, firms build social networks, binding stakeholders together [49]. The formal and informal institutional contexts have different requirements for the legitimacies of enterprises. Consequently, establishing social relations with relevant stakeholders that represent the legitimacies of formal and informal institutions also means obtaining different relational capital for enterprises.

Compared with formal institutional relational capital, informal institutional relational capital may exert a more direct and powerful effect on executive human capital. The informal legitimacy system represents business cooperation by focal enterprises with the supply chain partners, scientific research institutions, and third-party social organizations. Most informal institutional relational capital comes from the individual social relations of senior executives, reducing the environmental uncertainties in decision making. The collaboration between enterprises and their partners can be promoted to increase the effectiveness [50] and accuracy of their decisions [51]. However, when faced with a large amount of informal institutional relational capital, executives may need to make more “concessions” to meet or cater to the legitimacies from different stakeholders. The existence of informal relational capital increases the likelihood that executives will be distracted from it. Scholars such as Coles and Li (2019) and Li (2021) [20,21] explored the informal relational capital between top-and-down in an organization, and provided a comprehensive understanding of these top executives and their roles in the bottom-up monitoring mechanism. Research findings of Coles and Li (2019) and Li (2021) [20,21] triggered possible theoretical dialogues of intricate relationship between externally informal social capitals and internal human capital which built up by top managers and by employees. From the perspective of optimal distinctiveness theory, an increase in informal institutional relational capital means that enterprise heterogeneity should be established through the human capital at the senior executive level to meet the legitimacies of the informal institutions of various stakeholders. Therefore, compared with formal institutional relational capital, the effect of executive human capital on organizational performance may be weakened or even reversed under the regulation of informal institutional relational capital. Thus, Hypothesis 2a was proposed:

**Hypothesis** **2a** **(H2a).** *Compared with formal relational capital, an increase in informal relational capital to some extent turns a positive relationship between executive human capital and performance into a negative one.*

Formal institutional relational capital can inevitably affect the process in which all employees use their knowledge, experience, and skills to create value for the enterprise [14,17]. However, employees in different departments always utilize their skills and knowledge with different goals.

In contrast, informal institutional relational capital may have a more specific impact on reaching the heterogeneous advantages of human capital and improving performance. The more informal the institutional stakeholders are (i.e., the more informal the institutional relational capital is), the more diverse and complex the legitimacy requirements of the informal institutional stakeholders are for the focal firm, and the more social resources the focal firm will obtain. These stakeholders will effectively promote both the employees’ explicit and implicit knowledge sharing and second-order innovation in different departments such that human capital at the employee level, including their professional skills, knowledge, and intangible assets, plays a greater role in cooperative relationships. Therefore, the constraints of the organizational boundary on human capital may be broken when combining with informal institutional relational capital. The effective combination of human capital at the employee level with informal institutional relational capital helps to further maximize the performance and heterogeneous advantages of enterprises. The optimal distinctiveness theory advocates a strategic balance between internal resources and external legitimate resources; the maximization of enterprise value is achieved by combining internal and external resources rather than the restriction of external legitimacy on internal heterogeneity. Therefore, based on optimal distinctiveness theory, it is assumed that informal institutional relational capital can help to break the inverted U-shaped effect of human capital at the employee level on performance to create greater value. Thus, Hypothesis 2b was proposed:

**Hypothesis** **2b** **(H2b).** *Compared with formal relational capital, a high level of informal relational capital enhances the inverted U-shaped relationship between human capital and performance.*

## 3. Data and Measurements

### 3.1. Data Collection

This study collected data from 113 publicly listed Chinese pharmaceutical corporations from 2001 to 2017 in multiple ways and through different channels. We obtained the financial and annual report data of sample enterprises from the China Stock Market and Accounting Research (CSMAR) Database to calculate the values of the human capital, structural capital, and control variables. The data for relational capital were collected through a content analysis of news about the sample enterprises for each year. The relevant news reports of the sample enterprises from 2001 to 2017 were collected through the financial news system of Chinese universities, based on which, their social activities with different stakeholders every year were screened out and coded. Based on this content analysis data, both formal and informal institutional relational capital for each sample enterprise each year were calculated. Financial data and content data about 1230 enterprises from annual reports and public media were collected.

### 3.2. Measures and Modeling

#### 3.2.1. Dependent Variable

Performance: This study adopted the financial performance (ROA) of each company as the indicator of firm performance to understand the relationship between internal intellectual capital and the firm’s market and financial value.

#### 3.2.2. Independent Variables

Employee human capital: In this study, the methods proposed by [16,52] were referred to when the value of the ratio of labor cost to income was calculated as an indicator of human capital at the employee level (EmployeeHC).

Executive human capital: Based on the measurement methods of [32,53] the education level of each executive member was coded to calculate the human capital of the executives (TMTHC). First, according to the method proposed by [32], the education level of each executive member was converted into the length of education: less than high school = 9 years, high school = 12 years, Bachelor’s = 16 years, Master’s = 18 years, and Ph.D. = 22 years. The overall executive human capital of the enterprise of that year can be obtained by summing the number of years of education that each executive received.

#### 3.2.3. Moderating Variable

Informal relational capital: By referring to the way informal relational capital is measured, as proposed by [13,32], this study coded and calculated the frequency that an enterprise established relations with different strategic stakeholders each year and the frequencies were set as the proxy variables of relational capital. Social connections of sample firms were categorized into five types: (1) links with supply chain partners, (2) links with third-party organizations (industrial associates or legal qualification institutions), (3) links with local governments, (4) links with universities, and (5) links with social media.

#### 3.2.4. Control Variables

The age and size of a firm: The age of a firm was calculated as the difference between the year its data were collected and its founding year. The size of firms was set as a control variable since large organizations are more likely to have the resources needed to adopt innovations [5,54] and to exploit existing knowledge [55]. The size of a firm was defined as the number of full-time employees [56].

For the implied cost of equity and debt capital, this study included analysts’ forecast dispersion as a control variable as they act as the proxies for the level of uncertainty perceived by financial analysts. Scholars assume a negative correlation between these cost of finance proxies and the dispersion in the financial analysts’ earnings forecasts [57,58,59]. Referring to the research by [51] in which the company’s level of indebtedness was applied, the proxy of the debt ratio, which was computed as total debt over total assets, leverage (debt-to-equity ratio), and information asymmetry (percentage of stock not held by the 10 largest shareholders of a firm) were used [60].

A firm’s performance is influenced by managerial tradition and organizational slack [61]. Thus, the organizational inertia was also set as a control variable, which was measured using the natural logarithm of previous profits and slack, as well as the firm’s liquidity indicator, which was measured as current assets over current liabilities [51].

Based on the collected data (the codes of all variables and the measurements are all summarized in Table 1, Table 2 and Table 3 demonstrate the correlational analysis results), this study selected the semi-logarithmic model to construct a regression model. The natural logarithm of ROA was calculated before the statistical software package Stata 13 was used for the general linear regression (with human capital being an independent variable and executive human capital being the independent variable) to verify the aforesaid hypothesis. We also checked the robustness of the model by testing the hypothesis with the following alternatives: (1) we set ROA to be lagged by two years so that the causality between independent variables and dependent variables was strengthened, (2) we applied both the general linear regression with a semi-logarithmic model and the panel regression fixed (firm and year) model so that the unobserved characters of firm and year were covered, and (3) we switched the control variable size to total assets to provide another measurement of each firm’s scale. The results of robustness are presented in Table A1, Table A2, Table A3 and Table A4 in Appendix A.

## 4. Results

The results are reported in Table 2, Table 3, Table 4 and Table 5, where Table 3 indicates that the correlation between control variables, such as liquidity and leverage, was high. To ensure the multicollinearity among the control variables and independent variables, we calculated the VIF of all control variables and independent variables, and the highest VIF was 1.0; therefore, the possibility of multicollinearity in our model was low. The major regression results are shown in Table 4 and Table 5. The research results shown in Table 4 mainly validated H1a and H2a, which were both supported, while the research results in Table 5 validated H1b and H2b. In Table 4, the results from model 1 show that human capital at the executive level had a significantly positive effect on *performance* (coef. = 0.08045, *p* = 0.051). Thus, human capital at the executive level had a positive impact on performance, and therefore, H1a was supported. The results of model 2 show that the interaction term between human capital at the executive level and informal relational capital had a significant negative effect on performance (coef. = −0.01184, *p* = 0.003). Thus, informal relational capital weakened the positive impact of human capital on performance at the executive level, and therefore, H2a was supported.

In Table 5, the results from model 3 show that *human capital at the employee level* had a slight positive effect on *performance* (coef. = 0.01680, *p* = 0.096). Furthermore, the results from model 4 show that *human capital at the employee level* had a significantly positive effect on *performance* (coef. = 0.08710, *p* < 0.001), while the *square of human capital at the employee level* had a significant negative effect on *performance* (coef. = −0.0126, *p* = 0.002). Thus, human capital at the employee level had an inverted U-shaped impact on performance, and therefore, H1b was supported. The results from model 5 show that the interaction term between the square of human capital at the executive level and informal relational capital had a significant positive effect on performance (coef. = 0.00396, *p* = 0.018). Thus, informal relational capital weakened the inverted U-shaped impact of human capital at the employee level on performance. There is an opposite impact against H2b; the moderation of informal relational capital is turning the inverse-U influence of employeeHC on performance into positive way, especially when the employeeHC demonstrated to be in a higher level, which independently has a negative effect on performance.

## 5. Discussion

This study found that human capital at both levels had a different influence on organizational performance. Specifically, human capital at the executive level had a monotonic positive effect on performance, while human capital at the employee level exerted an inverted U-shaped influence on performance. Our study highlighted the differences in the effects of human capital at both levels on organizational performance. Top managers impacted performance with their cognition and risk-taking based on upper echelon theory, while the effect of human capital at the employee level on performance was affected by labor productivity and efficiency and constrained by the boundary of organizational scale.

Based on optimal distinctiveness, this study argued that different types of relational capital with stakeholders representing different kinds of institutional legitimacy exert varied effects on the formation of internal heterogeneous resources, thus producing different combinations of internal and external resources to achieve optimal distinctiveness or maximize performance. It was found that, compared to formal institutional relational capital, informal institutional relational capital significantly moderated the effects of human capital at both levels of performance. Furthermore, informal institutional relational capital played a moderating role in the influence of human capital at both levels on performance in different ways. This result shows that with the increase in informal institutional relational capital, human capital at the executive level that promoted performance experienced a turning point due to the constraint of interpersonal relationships in terms of its impact on performance, while human capital at the employee level, limited by the boundary of organizational scales, may break through and optimize performance by integrating social resources and informal institutional legitimacy. The interacting influences of human capital at both levels on performance can be used to produce dynamic orchestrations with informal institutional relational capital at different levels to achieve dynamic optimal heterogeneity.

To sum up, the essential contribution of this study lies in its exploration of the dynamic coordination between human capital at both levels and informal institutional relational capital to achieve optimal distinctiveness. This finding deepens the current understanding of intellectual capital and explains the differences in the strategic actions of enterprises with heterogeneous resources concerning intellectual capital.

## 6. Conclusions

### 6.1. Findings

This research aimed to explore how the human capital at different levels and informal relational capital can be orchestrated to achieve heterogeneous advantages and boost performance. The data from China’s listed pharmaceutical companies from 2001 to 2017 revealed that human capital at the executive level led to a monotonic increase in performance; it was regulated by informal institutional relational capital in a negative manner. In addition, human capital at the employee level had an inverted U-shaped effect on performance. On the other hand, the regulation of informal institutional relational capital could overcome the scale boundary of enterprise human capital.

It was found that internal and external resources had two kinds of interactions with the three components of intellectual capital. First, human capital at both the employee and executive levels exerted an impact on performance in the same direction at first, and then in different directions. In this respect, enterprises need to maintain a certain balance in the orchestration of human capital at both levels to maximize their performance. Second, with the change in informal institutional relational capital, it is necessary for enterprises to dynamically orchestrate human capital at both levels to maintain their best performance. The orchestration of these two groups shows that the effect of human capital at both levels on performance will cross over and reverse due to its interaction with external informal institutional relational capital.

### 6.2. Implications

This research contributes to theoretical and practical implications. First, the test results of this study revealed that human capital at higher and lower levels of organizations had a distinguished influence on performance, furthering the theoretical understanding of human capital [16,17]. Second, this research investigated the moderating influence of informal relational capital between human capital and performance, combining the informal institutional theory and the relational capital in intellectual capital theory. Finally, because we proposed our hypotheses from the perspective of optimal distinctiveness theory, we built connections between the optimal distinctiveness mechanisms and the components of intellectual capital, which supports future research on intellectual capital.

This research revealed the mechanism of how human and relational capitals combine to help to improve the optimal distinctiveness and performance of knowledge-intensive companies, such as pharmaceutical firms. In practice, this finding may be helpful in strategic decision making about intellectual capital and management model design. According to our research, enterprises need to monitor human capital at both levels at the same time and evaluate the effects of their integrations with external informal relational capital on performance. This study recommends that investment in human capital at both levels should be adjusted when the relationship with informal institutional stakeholders changes to promote their integration with external stakeholders.

### 6.3. Limitation

This study has some limitations. First, the results exclude the effects of structural capital on intellectual capital. Second, the samples in this research only covered listed pharmaceutical companies in which the innovation and knowledge flows were not as active as those in SMEs. Thus, further studies are needed to test our conclusions with samples of SMEs. Third, knowledge acquisition and innovation in different industries have their own unique characteristics. The pharmaceutical sector is characterized by a huge investment in R&D in the early stages with a low likelihood of knowledge spillover. Subsequent studies should also investigate other industries that are active in knowledge spillover and second-order innovation so that the results of this study can be compared and contrasted with those of future studies.

### 6.4. Future Directions for Research

Discussions of intellectual capital theory include human capital and structural capital within an enterprise, as well as relational capital outside the enterprise. The crossover mechanisms between its various components and the rules of resource arrangement are constantly being emphasized as the ongoing innovations in various industries around the world emerge one after another in the coming era. This study was dedicated to disclosing the different mechanisms of human capital on performance under the influence of external informal relational capital at different organizational levels, and thus to further explore the interactive effects of various antecedents of intellectual capital at different organizational levels for future practical use and thus build up the foundation for theoretical development. On the other hand, adhering to the leader–member exchange (LMX) theoretical perspective, scholars have explored both the informal relationship between employees at different levels in the enterprise and its impact on corporate performance in recent years (Coles and Li, 2019; Li, 2021) [20,21], and thus triggered the externally informal social capitals and internal human capitals’ possible theoretical dialogues. Future research can be developed toward a focus on the theoretical themes of human capital, which may expand the interaction between internal and external (formal or informal) relational capital of the enterprise and its role in improving the strategic performance of human capital.

## Figures and Tables

**Table 1:** Coding and measurement of the variables.

Variables	Measure
Dependent variables	
ROA	Return of assets
Independent Variables	
Executive human capital (TMTHC)	Years of education received: less than high school = 9 years, high school = 12 years, Bachelor = 16 years, Master = 18 years, and Ph.D. = 22 years
Employee human capital (EmployeeHC)	Employees’ salaries and expenses
Moderating variables	
Informal relational capital (InforRC)	Frequency of relations with supply chain partners, university or research institutes, and other NPOs
Control variables	
Size	Number of employees
Age	Number of years between the founding year and the year in which the firm was studied
Debt ratio	Total debt over total assets
Leverage	Debt-to-equity ratio
Asymmetry	Percentage of stock not held by the 10 largest shareholders of a firm
Liquidity	Current assets over current liabilities
Inertia	Natural logarithm of previous profits

**Table 2:** Summary of variables.

	Obs	Mean	Std. Dev.	Min	Max
Liquidity	1230	3.68892	7.742735	0.189947	190.8692
Debt_ratio	1230	0.35867	0.2159784	0.007521	1.893078
Leverage	1230	3.892685	6.85544	−0.47176	131.9557
Age	1230	13.32439	5.528061	0	36
Size	1192	3558.762	4054.906	20	28,848
Asymmetry	1230	0.609417	1.354812	0	47.67
Inertia	1121	16.46855	8.472025	−20.4401	21.92827
Employee HC (millionRMB)	1230	38.9	84.7	−3.373657	800
TMTHC	1230	16.23921	1.527277	11.16667	20
InforRC	1230	3.296748	25.44112	0	833
ROA	1230	0.062279	0.0750448	−0.411759	0.46404

**Table 3:** Correlation analysis results.

	Liquidity	Debt_ratio	Leverage	Age	Size	Asymmetry	Inertia	employeeHC	TMTHC	InforSC	ROA
Liquidity	1.00										
Debt_ratio	−0.34	1.00									
Leverage	0.94	−0.47	1.00								
Age	−0.04	0.06	−0.06	1.00							
Size	−0.12	0.18	−0.17	0.10	1.00						
Asymmetry	0.18	−0.20	0.17	0.17	0.11	1.00					
Inertia	0.09	−0.35	0.11	−0.01	0.14	0.15	1.00				
employeeHC	−0.06	0.07	−0.08	−0.02	0.63	0.15	0.12	1.00			
TMTHC	0.04	0.14	0.01	0.20	0.14	0.06	0.00	0.10	1.00		
InforRC	0.00	−0.07	0.01	0.15	0.07	0.10	0.12	0.00	0.08	1.00	
ROA	0.13	−0.27	0.21	0.01	−0.01	0.17	0.21	0.00	0.02	0.11	1.00

**Table 4:** General linear regression for executive human capital and ROA.

	Model 1 (H1a)			Model 2 (H2a)		
	coef.	t	*p* > |t|	coef.	t	*p* > |t|
_cons	−6.23803	−9.62	0.000	−6.59626	−10.1	0.000
Liquidity	−0.02746	−1.15	0.251	−1.10000	−1.17	0.244
Debt_ratio	−2.42055	−7.13	0.000	−2.34750	−6.94	0.000
Leverage	0.04919	1.69	0.092	0.04983	1.72	0.086
Age	−0.02333	−2.04	0.041	−0.02703	−2.3	0.020
Size	0.00001	0.47	0.640	0.00001	0.07	0.947
Asymmetry	0.02354	0.54	0.586	0.02360	0.55	0.583
TMTHC	0.08045	1.95	0.051	0.10239	2.47	0.014
InforRC				0.21825	2.86	0.004
TMTHC × inforRC				−0.01184	−2.92	0.003
No. of Observations	1174			1174		
Wald chi^2^	114.6			128.26		
ΔWald chi^2^				13.66		
Prob > chi^2^	0.000			0.000		
Log-likelihood	−2504.4867			−2498.296		

**Table 5:** General linear regression for employee human capital and ROA.

	Model 3			Model 4 (H1b)			Model 5 (H2b)		
	coef.	t	*p* > |t|	coef.	t	*p* > |t|	coef.	t	*p* > |t|
_cons	−5.82131	−19.29	0.000	−5.76049	−19.13	0.000	−5.76049	−19.13	0.000
Liquidity	−0.03995	−1.56	0.118	−0.03878	−1.52	0.128	−0.03924	−1.55	0.122
Debt_ratio	−1.77328	−4.49	0.000	−1.75967	−4.47	0.000	−1.72167	−4.38	0.000
Leverage	0.07311	2.26	0.024	0.07215	2.24	0.026	0.07305	2.27	0.023
Age	−0.01422	−1.19	0.234	−0.02344	−1.92	0.056	−0.02538	−2.04	0.042
Size	−0.00002	−0.97	0.334	−0.00004	−1.76	0.079	−0.00004	−1.85	0.065
Asymmetry	0.01906	0.44	0.663	0.02077	0.48	0.634	0.02049	0.47	0.637
Inertia	0.03077	3.76	0.000	0.02815	3.44	0.001	0.02759	3.37	0.001
employeeHC	0.01680	1.67	0.096	0.08710	3.54	0.000	0.09920	3.98	0.000
InforSC							0.01622	1.58	0.114
employeeHC^2^				−0.01260	−3.13	0.002	−0.00126	−3.13	0.002
employeeHC × inforRC							−0.00235	−2.22	0.027
employeeHC^2^ × inforRC							0.00396	2.38	0.018
N. Obs	1085			1085			1085		
F	15.09			14.62			11.88		
Prob > F	0.000			0.000			0.000		
R-squared	0.1009			0.109			0.1174		
Adj R-squared	0.0942			0.1016			0.1075		
ΔAdj R-squared				0.0074			0.0059		

## Appendix A

**Table A1:** Results of the panel regression with random effects.

	Model 1	Model 2
	F2LnROA	F2LnROA
TMTHC	0.144 ***	0.165 ***
	(2.65)	(2.82)
InforRC		0.184
		(0.56)
TMTHC_InforRC		−0.015 *
		(−0.77)
Totalasset (firm scale)	−0.000 **	−0.000 **
	(−2.17)	(−1.99)
age	0.012	0.016
	(0.67)	(0.91)
Debt_ratio	−0.414	−0.434
	(−1.07)	(−1.12)
Leverage	0.008 **	0.008 **
	(2.38)	(2.38)
Asymmetry	1.499 ***	1.574 ***
	(2.89)	(3.03)
Liquidity	0.009	0.008
	(0.98)	(0.91)
_cons	−9.120 ***	−9.471 ***
	(−10.13)	(−9.77)
Wald chi^2^	657.66	665.29
ΔWald chi^2^		7.63
Prob > chi^2^	0.0000	0.0000
N	965.000	965.000

*t* statistics in parentheses; * *p* < 0.1, ** *p* < 0.05, *** *p* < 0.01.

**Table A2:** Results of the panel regression with random effects.

	Model 3	Model 4	Model 5
	F2LnROA	F2LnROA	F2LnROA
EmployeeHC	5.994 *	0.274	2.846
	(1.49)	(0.03)	(0.31)
InforRC			−0.065
			(−1.15)
EmployeeHC_2		43.722 **	29.532
		(0.84)	(0.53)
EmployeeHC_2_InforRC			25.199 *
			(0.32)
EmployeeHC_InforRc			−2.330
			(−0.44)
Totalasset (firm scale)	−0.000 *	−0.000 *	−0.000 *
	(−1.95)	(−1.89)	(−1.66)
age	0.017	0.016	0.023
	(0.96)	(0.88)	(1.25)
Debt_ratio	−0.335	−0.399	−0.427
	(−0.76)	(−0.91)	(−0.96)
Leverage	0.008 **	0.008 **	0.008 **
	(2.24)	(2.27)	(2.22)
Asymmetry	1.560 ***	1.588 ***	1.665 ***
	(2.79)	(2.85)	(2.98)
Liquidity	0.009	0.008	0.008
	(0.91)	(0.85)	(0.80)
Intertia	0.001	0.002	0.003
	(0.16)	(0.22)	(0.42)
_cons	−7.073 ***	−6.988 ***	−7.062 ***
	(−13.18)	(−12.89)	(−13.00)
N	874.000	874.000	874.000
Wald chi^2^	533.51	558.01	583.81
Δwald chi^2^		24.5	25.8
Prob > chi^2^	0.0000	0.0000	0.0000

*t* statistics in parentheses; * *p* < 0.1, ** *p* < 0.05, *** *p* < 0.01.

**Table A3:** Results of the panel regression with fixed effects (firm and year).

	(1)	(2)
	F2LnROA	F2LnROA
TMTHC	0.164 **	0.165 **
	(2.44)	(2.38)
TMTHC_InforRC		−0.004 *
		(−0.18)
InforRC		−0.025
		(−0.07)
totalasset	−0.000 ***	−0.000 ***
	(−2.70)	(−2.59)
age	0.023	0.032
	(1.02)	(1.43)
Debt_ratio	0.621	0.603
	(1.38)	(1.34)
Leverage	0.008 **	0.008 **
	(2.37)	(2.33)
Asymmetry	1.147 *	1.246 **
	(1.90)	(2.06)
Liquidity	0.015	0.015
	(1.41)	(1.39)
_cons	−9.740 ***	−9.829 ***
	(−9.21)	(−8.84)
r^2^	0.031	0.038
Δr^2^		0.007
F	3.826	3.733
N	965.000	965.000

*t* statistics in parentheses; * *p* < 0.1, ** *p* < 0.05, *** *p* < 0.01.

**Table A4:** Results of the panel regression with fixed effects (firm and year).

	(3)	(4)	(5)
	F2LnROA	F2LnROA	F2LnROA
EmployeeHC	3.475	−2.043	2.885
	(0.80)	(−0.22)	(0.28)
InforRC			−0.068
			(−1.14)
EmployeeHC_2		37.468	10.566
		(0.67)	(0.18)
EmployeeHC_2_InforRC			83.184
			(0.98)
EmployeeHC_InforRc			−6.573
			(−1.15)
totalasset	−0.000 ***	−0.000 ***	−0.000 **
	(−2.61)	(−2.61)	(−2.34)
age	0.039 *	0.038 *	0.049 **
	(1.82)	(1.80)	(2.29)
Debt_ratio	0.655	0.650	0.637
	(1.29)	(1.28)	(1.26)
Leverage	0.008 **	0.008 **	0.008 **
	(2.23)	(2.26)	(2.19)
Asymmetry	1.055 *	1.068 *	1.197 *
	(1.65)	(1.67)	(1.88)
Liquidity	0.012	0.012	0.012
	(1.14)	(1.09)	(1.06)
Intertia	−0.002	−0.002	−0.000
	(−0.28)	(−0.26)	(−0.00)
_cons	−7.279 ***	−7.210 ***	−7.377 ***
	(−12.27)	(−11.97)	(−12.24)
r^2^	0.024	0.025	0.039
Δr^2^		0.001	0.014
F value	2.374	2.158	2.584
No. of Observations	874.000	874.000	874.000

*t* statistics in parentheses; * *p* < 0.1, ** *p* < 0.05, *** *p* < 0.01.
